# Unusual Presentation of Recurrent Early Stage Endometrial Carcinoma 28 Years after Primary Surgery

**DOI:** 10.1155/2015/256838

**Published:** 2015-12-09

**Authors:** Alessandro Franchello, Gianruggero Fronda, Giacomo Deiro, Alessia Fiore, Davide Cassine, Luca Molinaro, Luigi Chiusa, Sara Galati, Andrea Resegotti, Stefano Silvestri

**Affiliations:** ^1^4th General Surgery Department, Azienda Ospedaliera Universitaria Città della Salute e della Scienza, 10126 Turin, Italy; ^2^2nd Phathological Anatomy Department, Azienda Ospedaliera Universitaria Città della Salute e della Scienza, 10126 Turin, Italy

## Abstract

Endometrial carcinoma is the most common neoplasia of female genital tract. The prognosis of early stage disease (FIGO I and FIGO II) is excellent: recurrence after surgery is less than 15%, most of which are reported within 3 years after primary treatment. Herein we report a case of late rectal recurrence from FIGO Ib endometrial adenocarcinoma. Patient had also familiar and personal history of colonic adenocarcinoma and previous findings of microsatellite instability (MSI); molecular analysis evidenced heterozygotic somatic mutation in MLH1 gene. Twenty-eight years after hysterectomy and bilateral salpingoovariectomy, a rectal wall mass was detected during routine colonoscopy. Patients underwent CT scan, pelvic MRI, and rectal EUS with FNA: histopathological and immunohistochemical analysis revealed differentiated carcinoma cells of endometrial origin. No neoadjuvant treatment was planned and low rectal anterior resection with protective colostomy was performed; histology confirmed rectal lesion as metastasis from endometrial carcinoma. Recurrence of early stage endometrial carcinoma after a long period from primary surgery is possible. It is important to keep in mind this possibility in order to set a correct diagnostic and therapeutic algorithm, including preoperative immunohistochemical staining, and to plan a prolonged follow-up program.

## 1. Introduction

Endometrial carcinoma (EC) is the most common neoplasia of female genital tract: the highest incidence occurs in postmenopausal women (70%), whereas it is uncommon under 40 years of age (<5%) [1]. The prognosis of early stage disease (International Federation of Gynecology and Obstetrics (FIGO) I and FIGO II) is excellent, with a reported 5-year survival rate from 80% to more than 90% for FIGO Ib [2]; in advanced stages of disease, survival rate decreases to 20–25% (FIGO IV) [3]. Recurrence rate in early stage (FIGO I and FIGO II) is 15% [2, 4]; the majority recur within 3 years after primary treatment [2]; relapses after many years are quite rare in literature. We report a case of rectal recurrence from endometrial adenocarcinoma 28 years after first surgery.

## 2. Case Presentation

A 72-year-old woman was admitted to our department for the findings of rectal mass, in the absence of abdominal pain and alteration of the intestinal canalization, after a routinely endoscopic investigation in the context of oncological follow-up. The patient was previously submitted in 1985 to hysterectomy and bilateral salpingoovariectomy for early stage low-grade endometrioid EC (FIGO Ib) associated with Intravaginal Brachytherapy (IVBT), in 1989 to left thyroid lobectomy for papillary carcinoma followed by I^131^ adjuvant radiotherapy, in 2001 to endoscopic resection of bladder papillary carcinoma, and in 2003 to right hemicolectomy for pT3 N1 (1/23) colonic adenocarcinoma followed by adjuvant chemotherapy. Due to positive familiar and personal history of oncologic diseases, oncologists referred patient to genetic evaluation: microsatellite instability (MSI) by heterozygotic somatic mutation in MLH1 gene was detected.

In 2013 routine colonoscopy was performed and a right-sided compression of rectal wall with an unscathed mucosa was found; the patient was submitted to pelvic Magnetic Resonance Imaging (MRI) demonstrating a mass of 4.7 × 3.5 cm at 6.5 cm from the anocutaneous plane and about 3 cm from the anorectal junction.

**Figure 1:**
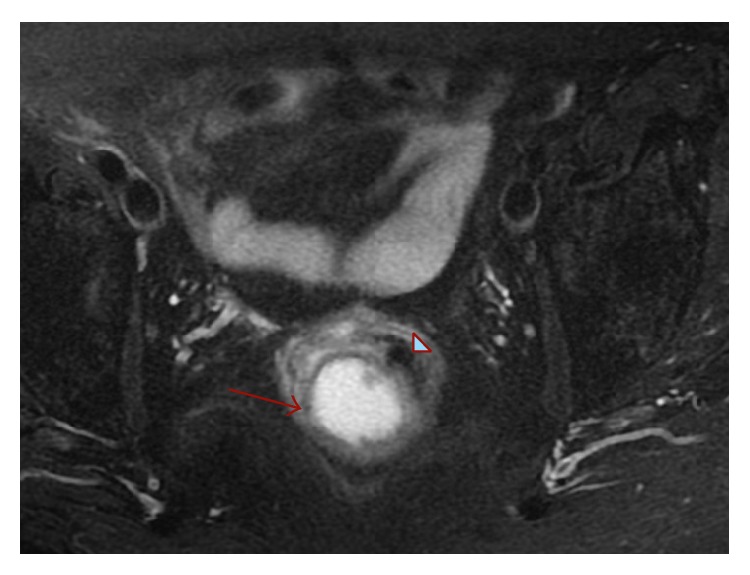
Pelvic MRI. Mass (red arrow) compressing intestinal lumen.

The mass had intramural development with compression of the gut lumen and without any pelviperineal muscular involvement. T1 and T2 weighted phases evidenced a small oval area (11 mm) in perivisceral adipose tissue with similar characteristics: a mesorectal lymphadenopathy with maximum diameter of 8 mm was also identified (Figure 1).

**Figure 2:**
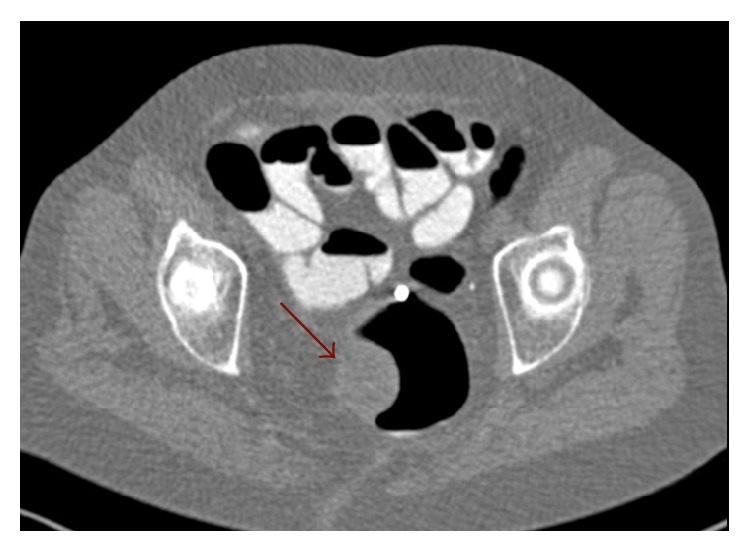
MDCT scan. Mass with slightly eccentric hypodense area and early enhancement after contrast medium intravenous injection.

Multidetector Computed Tomography (MDCT) confirmed a rectal hypodense lesion with a slightly eccentric hypodense area and medial component intensely enhanced in the arterial phase, with a single mesorectal lymph node: no liver or pulmonary metastasis was found (Figure 2).

**Figure 3:**
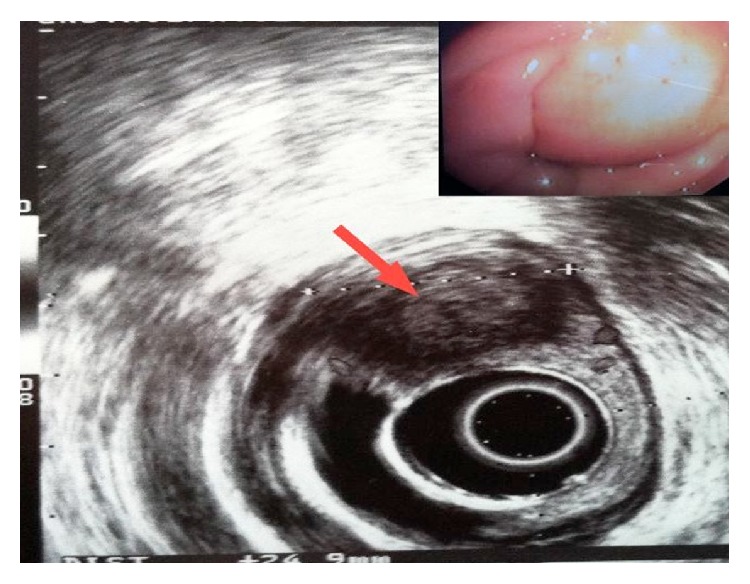
EUS. Subepithelial mass of 3 cm diameter with perirectal lymphadenopathy.

Endoscopic ultrasound (EUS) showed once more a subepithelial hypoechogenic mass in the right paramedian side of rectal wall (Figure 3), with absence of significant vascularization, and a lymphadenopathy with a maximum diameter of up to 6-7 mm.

Fine needle aspiration biopsy (FNAb) was performed on rectal wall mass (multiple aspiration with Procore 22-gauge needle).

**Figure 4:**
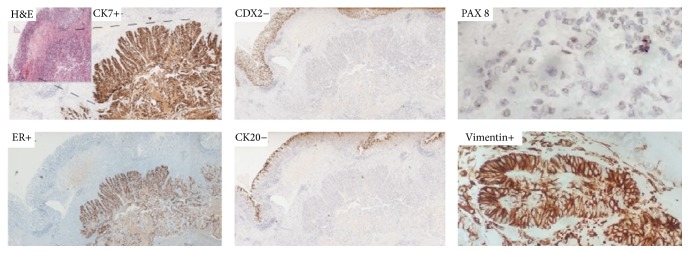
Immunohistochemistry. Hematoxylin and eosin stain (H&E stain) with unharmed mucosa (arrow), CK7+, ER+, CDX2−, CK20−, antivimentin Ab+, and PAX 8+ (focally).

At cytohistology poorly differentiated carcinoma cells were found with immunocytochemical profile of endometrial origin in CEA (+), CDX2 (−), CA125 (−), and estrogen receptors (+). No endometriosis foci were found in all the specimens analyzed. Sieric neoplastic markers were negative. After multidisciplinary discussion, no neoadjuvant treatment (radiotherapy (RT), chemotherapy (CT), or CT + RT) was proposed, and indication for rectal resection was given. At laparotomy, a single hepatic metastasis in the segment 3 of 8 mm was found. After liver Intraoperative Ultrasound (IOUS), which confirmed absence of other focal lesions, hepatic wedge resection and rectal anterior resection associated with protective colostomy were performed. Postoperative course was uneventful and patient was discharged on 6th p.o. day. Histological examination confirmed rectal mass as a metastatic adenocarcinoma, not a primary rectal cancer, with immunohistochemical features of endometrial carcinoma CK7+, ER+, CDX2−, CK20−, antivimentin Ab+, and PAX 8+ (focally) (Figure 4).

All antibodies used were rabbit monoclonal antibody (Ventana Medical Systems, Inc., Tucson, AZ). CK7 (clone SP52), CK20 (clone SP33), vimentin (clone V9), CA125 (clone 8C125), and estrogen receptors (clone SP1) were prediluted antibody; CEA (clone TF3H8-1) and CDX2 (clone EPR2764Y) were diluted 1 : 1. The slides were stained with these antibodies using the Ventana automated immunostainer (BenchMark AutoStainer; Ventana Medical Systems, Tucson, AZ, USA). The hepatic and perirectal lesions evidenced the same immunohistochemical pattern. Invasion from endometrial adenocarcinoma in a single perirectal lymph node out of twelve was documented. After discharge, the revision of histological slides of patient's initial intervention was done: endometrial tumor removed 28 years before was confirmed to be a primary low-grade endometrioid EC with the same histopathological features. No indications for adjuvant treatment were done and clinical/radiologic follow-up was planned. After eight months the colostomy was closed and 23 months later the patient was disease-free.

## 3. Discussion

Among histological types of uterus body cancers, endometrial adenocarcinoma is the most frequent; it is found in 75% of cases and it is the most common gynaecological neoplasia in Western countries. Large majority of endometrial cancers are sporadic; genetic mutations cause endometrial cancer in about 5% of patients. Screening for genetic mutations should be considered in all patients with endometrial cancer, but especially in those younger than 50 years of age [1]. Overall rate of endometrial cancer recurrence is approximately 15% [5]; in more than 50% of cases, it develops within 2 years, and about 75% occurs within 3 years after primary treatment [6]. Relapse of early stage EC is extremely rare; to our knowledge, in literature there are only three case reports of patients with single recurrent disease directly involving the digestive tract after a period of clinical latency: Wou et al. [7] reported the occurrence of a gastrointestinal bleeding due to metastatic endometrial cancer 2 years after initial surgical treatment; Addison et al. [8], respectively, described a single case of rectal metastasis from previously resected endometrial carcinoma and an appendiceal metastasis 10 years after primary 12 treatment [9].

Until 2014, 14 cases of solitary splenic metastasis from endometrial adenocarcinoma after a long period of clinical latency were also reported [10], and several sites of recurrence outside from abdominal cavity were described: umbilical [11], retro molar pad [12], lung [13], scalp [14], and abdominal skin metastasis [15].

In our patient, already from the early diagnostic stages, the lesion appeared with unusual endoscopic and radiological features. It was a subepithelial tumor with normal mucosa; in addition, MRI and MDCT scan showed an intramural well-defined lesion giving evidence for stromal tumor and especially for a rectal gastrointestinal stromal tumor (GIST). However, it was important to rule out the primary rectal origin of the lesion because the patient already underwent colectomy for colonic adenocarcinoma and she was affected by MSI (a hypermutable phenotype caused by the loss of DNA mismatch repair activity, detected in about 15% of all colorectal cancers [16, 17]). As the establishment of tumor histology is often difficult before surgery, EUS-FNA was considered mandatory to plan correct therapeutic approach and to avoid unnecessary treatments.

Differential diagnosis was considered especially towards intestinal endometriosis, a rare but well-known identity; it often presents with cyclic intestinal symptoms of many years' duration (such as abdominal pain, bloody diarrhoea, rectal bleeding, and recurrent bowel subobstruction). In addition, patients with suspected intestinal endometriosis have undergone one or more surgical procedures aimed at treating endometriosis previously. Absence of endometriosis does not exclude completely the possibility of a primary endometrial carcinoma arising from an endometriotic focus but, in this case, it would be at least probable to find other endometriotic foci in the surrounding tissue.

Malignant transformation of endometriosis (EMT) is extremely rare and occurs in 1% of case; the association between development of malignancy and exogenous hormonal therapy is well known. EMT occurs electively in ovaries in more than 80% of patients, but gastrointestinal tract involvement has already been reported and classified as endometriosis-associated intestinal tumors (EAITs) [18]. Sampson postulated pathological criteria for assessment of EMT; tumor cells have typical müllerian characteristic resembling endometrial origin of cells; tumors are contiguous to or admixed with endometriotic foci localized in the bowel; neoplasia does not represent obvious metastasis from gonadal neoplasms and occurs typically in women from 30 to 60 years with signs and symptoms as abdominal or pelvic pain, pelvic mass, or vaginal bleeding [18–20]. According to Sampson's criteria, in the case reported EAIT was excluded.

In preoperative period, whenever possible, it is also important to use immunohistochemistry (IHC) to support difficult pathological differential diagnosis and to reduce the rate of false negative and false positive. Therefore it would be beneficial to integrate all of the current knowledge on IHC markers for the differential diagnosis of pelvic organs neoplasia. Carcinoma of pelvic organs presents distinct patterns of immunoreactivity for keratins (CK or KRTs) and it also expresses fundamental markers for differential diagnosis of different pelvic masses. From 2003, many reports document CDX2 and CK20 positivity in almost all rectal adenocarcinomas, but negativity in endometrial carcinoma [21]. In the clinical case presented the positivity to PAX 8, even if focal, supports müllerian origin [22] and it has been useful to confirm a nongastrointestinal (GI) primary origin as well as the strong vimentin positivity.

In conclusion, metastatic involvement of the GI tract from endometrial adenocarcinoma after a long period of clinical latency is a rare but possible occurrence. Consequently, it is important to keep in mind this possibility in order to set a correct diagnostic and therapeutic algorithm, including preoperative immunohistochemical staining. A prolonged clinical and instrumental follow-up program for high-risk cancer patients should always be performed.
